# Vesicocutaneous fistula due to vesical diverticulitis with stones: A case report and literature review

**DOI:** 10.1002/iju5.12546

**Published:** 2022-10-11

**Authors:** Jun Akatsuka, Kyota Suzuki, Shunsuke Ikuma, Masato Yanagi, Yuki Endo, Hayato Takeda, Yuka Toyama, Teruyuki Dohi, Go Kimura, Yukihiro Kondo

**Affiliations:** ^1^ Department of Urology Nippon Medical School Tokyo Japan; ^2^ Department of Plastic, Reconstructive and Aesthetic Surgery Nippon Medical School Tokyo Japan

**Keywords:** calculi, diverticulum, urinary bladder, vesicocutaneous fistula

## Abstract

**Introduction:**

We encountered an extremely rare case of a vesicocutaneous fistula due to vesical diverticulitis with stones.

**Case presentation:**

A 78‐year‐old male patient presented to our department with complaints of suppurative discharge in the suprapubic area. Computed tomography revealed an enlarged prostate, a vesical diverticulum with stones located on the ventral side, and an aberrant connection between the anterior bladder wall and the external surface of the skin. The patient was diagnosed with a vesicocutaneous fistula due to vesical diverticulitis and was successfully treated with a multidisciplinary approach including vesical diverticulectomy with stone removal and nonviable tissue debridement. The patient continues to receive regular outpatient follow‐ups with urinary catheter changes.

**Conclusion:**

Vesicocutaneous fistulas due to vesical diverticulitis with stones are extremely rare. We should be aware that a vesical diverticulum with stones located on the ventral side might pose a high‐risk factor for the formation of a vesicocutaneous fistula in elderly patients.

**Keynote message**

We report an extremely rare case of a vesicocutaneous fistula due to vesical diverticulitis with stones in an elderly patient. The case findings highlight the importance of considering the possibility of elderly patients developing vesicocutaneous fistulas due to a vesical diverticulum with stones located on the ventral side.

## Introduction

Vesicocutaneous fistulas are abnormal tracts formed between the urinary bladder and the cutaneous surfaces of the body. Although rare, they have been reported after trauma, surgery, and radiation therapy on the pelvic area.^1^ Vesicocutaneous fistulas might be due to bladder wall inflammation caused by vesical stones, but few cases have been reported to date in the literature. Herein, we describe an extremely rare case of a vesicocutaneous fistula due to vesical diverticulitis with stones in an elderly male patient.

## Case presentation

A 78‐year‐old man presented to our department with complaints of suppurative discharge in the suprapubic area, intermittent dysuria, and a feeling of fullness in the lower abdomen for a few years.

**Fig. 1:**
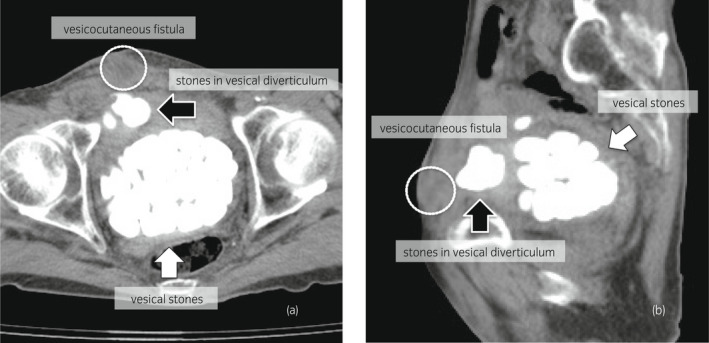
Abdominal computed tomography images (a: axial; b: sagittal) demonstrating vesical calculi (white arrows), a vesical diverticulum calculus (black arrow), and abnormal changes suspected to indicate a vesicocutaneous fistula (white circle).

**Fig. 2:**
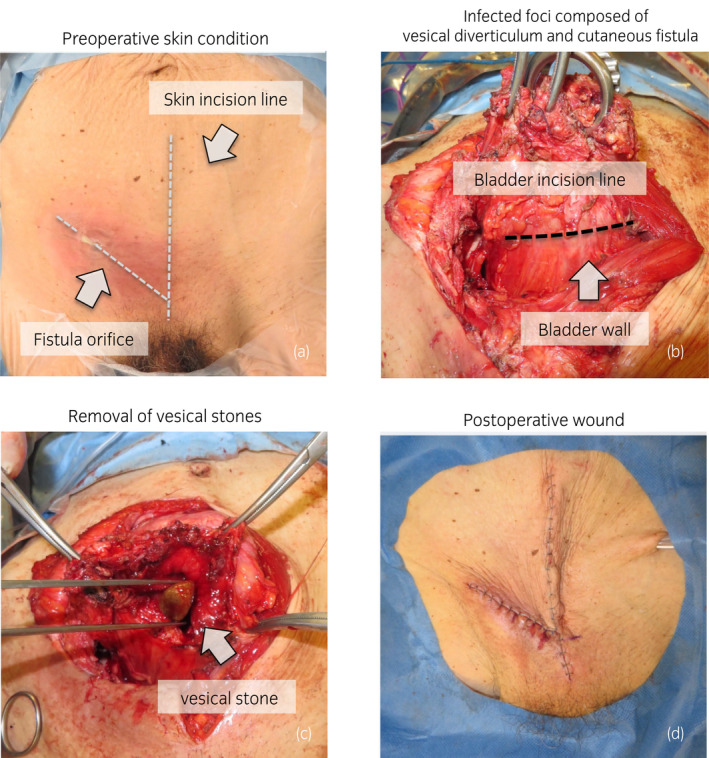
Surgical procedure used in this case. (a) We approached the space of Retzius via a midline incision in the lower abdomen and an incision centered on the fistula in the lower right abdomen. (b) The infected wound including the fistula and the vesical diverticulum were removed en bloc. (c) After releasing the bladder, all the vesical calculi were removed. (d) The infected tissues were debrided and the incisions were closed.

**Fig. 3:**
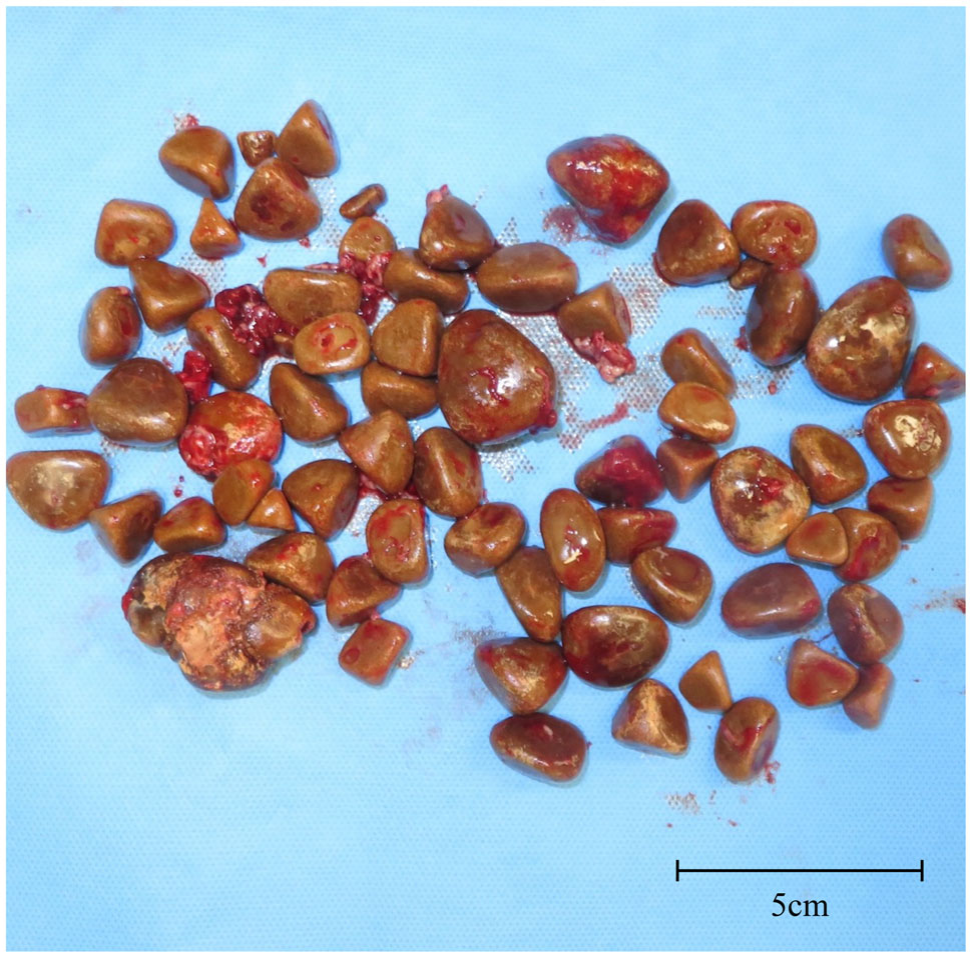
Photograph showing multiple vesical diverticulum calculi and vesical calculi (maximum diameter: 4.1 cm).

Skin redness with tension was observed in the lower right abdomen. Fluid discharge was observed from the center of the reddish region. His performance status was poor (Karnofsky Performance Status: 60%) due to previously untreated chronic obstructive pulmonary disease with long‐term effects from smoking. On the other hand, the patient had no medical history that would predispose him to a neurogenic bladder. Blood tests revealed a mild inflammatory response (white blood cells, 6000/μL; C‐reactive protein, 1.66 mg/dL). The pus culture test from the fistula revealed the presence of *Staphylococcus aureus* and *viridans* group streptococci. Computed tomography revealed multiple vesical stones (maximum diameter: 4.1 cm), and vesical diverticulum with stones located on the ventral side as well as an aberrant connection between the anterior bladder wall and the external surface of the skin, which was thought to be caused by the enlarged prostate (calculated volume: 79 mL; Fig. 1ab). Based on the patient's medical history and imaging findings, the lesion was diagnosed as a vesicocutaneous fistula due to vesical diverticulitis with stones. We performed an open surgery with the patient under general anesthesia. To secure an adequate field of view and to remove the infectious tissue en bloc, we made a midline incision in the lower abdomen and an oblique incision centered on the opening of the fistula, following the advice of the reconstructive surgery team (Fig. 2a). The infection appeared to be localized around the fistula in the abdominal wall. To control infection, the abdominal wall tissues around the fistula and the vesical diverticulum were removed en bloc, and we retained as much normal tissue as possible (Fig. 2b). The vesical stones were also removed (Fig. 2c). The inside of the bladder was washed with saline, and the bladder wall was sutured (Fig. 2d). We removed 72 stones, which were brown‐colored and had a maximum diameter of 4.1 cm (Fig. 3). Analysis revealed that the stones were composed of magnesium ammonium phosphate. The patient received antibiotic therapy (ceftriaxone sodium hydrate) for preventing surgical site infection, and the postoperative period was uneventful. Then, we administered an alpha‐1 blocker and dutasteride to the patient, but it was deemed difficult to make it catheter‐free. We recommended him transurethral surgery for the enlarged prostate as his next treatment; however, the patient preferred treatment with a urinary catheter to surgical therapy or clean intermittent catheterization. Currently, the patient is undergoing regular outpatient follow‐ups with urinary catheter changes.

## Discussion

Vesicocutaneous fistulas are abnormal tracts formed between the bladder and the cutaneous surfaces of the body. These are caused by congenital diseases as well as complications of surgery and radiation therapy.^1^ Vesicocutaneous fistulas are also known to be serious complications of vesical stones, but few cases have been reported to date in the literature.^2^, ^3^, ^4^, ^5^, ^6^, ^7^, ^8^, ^9^

We have summarized the clinical courses of 8 cases previously reported in the literature and that of our case in Table 1.^2^, ^3^, ^4^, ^5^, ^6^, ^7^, ^8^, ^9^ The median age of patients with vesicocutaneous fistulas caused by vesical stones was 60 years (range: 30–80 years), and most patients were male (7/9 cases; 77.8%). The formation of vesical diverticulitis with stones is associated with bladder wall fragility, and these patients in particular had a medical history of trauma, surgery, prostate hyperplasia, and neurogenic bladder. In most cases, the orifice of the fistula was located in the area from the abdominal wall to the groin. One case was reported with complaints of continuous fluid drainage from the perineum in a patient whose medical history began 12 years ago as a perianal abscess.^9^ The vesical stones were composed of magnesium ammonium phosphate (3/4 cases; 75.0%), suggesting the presence of severe urinary tract infection. Our patient had many magnesium ammonium phosphate stones, caused by a long‐term infectious condition in the bladder. Controlling urinary tract infections and residual urine that cause vesical stones can prevent the formation of a vesicocutaneous fistula.

**Table 1:** Reported cases of vesicocutaneous fistula caused by vesical calculus

No.	Year	Author	Age	Sex	Location of orifice	Factors associated with dysuria	History of trauma or surgery	Treatment	Stone analysis
1	1992	Motiwala et al.^2^	30	Male	Midway between umbilicus and symphysis pubica	–	Cystolithotomy 5 years before	Cystolithotomy and excision of the fistula	Mixed stone containing calcium, phosphate, oxalate, and uric acid
2	2005	Kishore et al.^3^	55	Male	Left groin	Vesical diverticulum	Drainage of an abscess at the left groin	Diverticulectomy and excision of the fistulous tract	Unknown
3	2007	Kobori et al.^4^	62	Female	Lower abdomen	–	Ovariectomy 20 years before	Cystolithotomy followed by closure of fistula	Magnesium ammonium phosphate
4	2010	Horsnell et al.^5^	62	Female	Right groin	Myelomeningocele spina bifida	–	Cystolithotomy and the fistula tract left to close spontaneously	Unknown
5	2011	Deshmukh et al.^6^	60	Male	Lower abdomen	–	Cystolithotomy 5 years before	Cystolithotomy and removal of giant calculus	Unknown
6	2012	Cavazzoni et al.^7^	80	Male	Abdominal wall	Severe prostate hyperplasia	–	Cystolithotomy and removal of giant stones	Unknown
7	2016	Goumas et al.^8^	56	Male	Midway between the umbilicus and pubic symphysis	–	Abdominal operation because of a severe car accident	Cystolithotomy and fistula repair	Magnesium ammonium phosphate
8	2018	Ahmed et al.^9^	52	Male	Perineum	–	Perineal abscess	Cystolithotomy and the fistula tract left to close spontaneously	Unknown
Our case	2022	Akatsuka et al.	78	Male	Lower right abdomen	Benign prostatic hyperplasia and vesical diverticulum	–	Diverticulectomy, excision of the fistula, and nonviable tissue debridement	Mixed stone based on magnesium ammonium phosphate

Surgical treatment is essential to cure this disease, and surgeries were performed in all reported cases. The purpose of the surgery is to control the infectious condition by removing the structures associated with the vesical stones and fistula. In the present case, the vesical diverticulum with stones were considered a contributing factor, and we performed multidisciplinary treatment including vesical stone removal, bladder diverticulectomy, and nonviable tissue debridement via an en bloc approach. Surgical wound dehiscence is one of the common complications after cystolithotomy. To prevent the occurrence of this complication, we cleaned the inside of the bladder with a large amount of saline, removed the infectious tissues, and retained as much normal tissue as possible.

Vesical diverticulum formation occurs secondary to bladder outlet obstruction, which develops as herniations of the bladder wall between the defects of smooth muscle fibers.^2^ A vesical diverticulum can cause a variety of complications such as residual urine, urinary tract infections, and diverticular stones.^10^ The possible mechanism of development of this condition in our case is as follows. Bladder outlet obstruction due to an enlarged prostate (calculated volume: 79 mL) led to increased intravesical pressure, vesical pseudodiverticulum formation, persistent residual urine, urinary tract infection, and multiple magnesium ammonium phosphate stone formation. The inflammation, which spread to the abdominal wall near the ventral vesical diverticulum, resulted in a vesicocutaneous fistula.

In conclusion, we report an extremely rare case of a vesicocutaneous fistula caused by vesical diverticulitis with stones in an elderly male patient. We should be aware that vesical diverticular stones located on the ventral side might pose a high‐risk factor for the formation of a vesicocutaneous fistula in elderly patients.

## Author contributions

Jun Akatsuka: Conceptualization; data curation; investigation; methodology; project administration; writing – original draft; writing – review and editing. Kyota Suzuki: Data curation; writing – original draft. Shunsuke Ikuma: Data curation. Masato Yanagi: Data curation. Yuki Endo: Data curation. Hayato Takeda: Data curation. Yuka Toyama: Data curation. Teruyuki Dohi: Data curation; investigation. Go Kimura: Supervision; writing – review and editing. Yukihiro Kondo: Supervision; writing – original draft; writing – review and editing.

## Conflict of interest

The authors declare no conflict of interest.

## Approval of the research protocol by an Institutional Reviewer Board

The ethics committees of Nippon medical school hospital gave approval for this study (#30‐03‐1099) and written informed consent was obtained in accordance with the World Medical Association Helsinki Declaration.

## Informed consent

Written informed consent was obtained from the patient for publication of this case report and any accompanying images.

## Registry and the Registration No. of the study/trial

Not applicable.

## Data Availability

The datasets analyzed during the current study are available from the corresponding author upon reasonable request.
